# Renal tumor segmentation, visualization, and segmentation confidence using ensembles of neural networks in patients undergoing surgical resection

**DOI:** 10.1007/s00330-024-11026-6

**Published:** 2024-08-23

**Authors:** Sophie Bachanek, Paul Wuerzberg, Lorenz Biggemann, Tanja Yani Janssen, Manuel Nietert, Joachim Lotz, Philip Zeuschner, Alexander Maßmann, Annemarie Uhlig, Johannes Uhlig

**Affiliations:** 1 Department of Clinical and Interventional Radiology, University Medical Center Goettingen, Goettingen, Germany; 2 Department of Medical Bioinformatics, University Medical Center Goettingen, Goettingen, Germany; 3 Department of Cardiac Radiology, University Medical Center Goettingen, Goettingen, Germany; 4 Department of Urology and Pediatric Urology, Saarland University, Homburg, Germany; 5 Department of Radiology & Nuclear Medicine, Robert-Bosch-Krankenhaus, Bosch Health Campus, Stuttgart, Germany; 6 Department of Urology, University Medical Center Goettingen, Goettingen, Germany; 7 Campus Institute for Data Science (CIDAS), Section of Medical Data Science (MeDaS), University of Goettingen, Goettingen, Germany

**Keywords:** Semantic segmentation, Kidney tumor segmentation, Renal tumor segmentation, Deep learning tumor segmentation, Ensembles of neural networks

## Abstract

**Objectives:**

To develop an automatic segmentation model for solid renal tumors on contrast-enhanced CTs and to visualize segmentation with associated confidence to promote clinical applicability.

**Materials and methods:**

The training dataset included solid renal tumor patients from two tertiary centers undergoing surgical resection and receiving CT in the corticomedullary or nephrogenic contrast media (CM) phase. Manual tumor segmentation was performed on all axial CT slices serving as reference standard for automatic segmentations. Independent testing was performed on the publicly available KiTS 2019 dataset. Ensembles of neural networks (ENN, DeepLabV3) were used for automatic renal tumor segmentation, and their performance was quantified with DICE score. ENN average foreground entropy measured segmentation confidence (binary: successful segmentation with DICE score > 0.8 versus inadequate segmentation ≤ 0.8).

**Results:**

*N* = 639/*n* = 210 patients were included in the training and independent test dataset. Datasets were comparable regarding age and sex (*p* > 0.05), while renal tumors in the training dataset were larger and more frequently benign (*p* < 0.01). In the internal test dataset, the ENN model yielded a median DICE score = 0.84 (IQR: 0.62–0.97, corticomedullary) and 0.86 (IQR: 0.77–0.96, nephrogenic CM phase), and the segmentation confidence an AUC = 0.89 (sensitivity = 0.86; specificity = 0.77). In the independent test dataset, the ENN model achieved a median DICE score = 0.84 (IQR: 0.71–0.97, corticomedullary CM phase); and segmentation confidence an accuracy = 0.84 (sensitivity = 0.86 and specificity = 0.81). ENN segmentations were visualized with color-coded voxelwise tumor probabilities and thresholds superimposed on clinical CT images.

**Conclusions:**

ENN-based renal tumor segmentation robustly performs in external test data and might aid in renal tumor classification and treatment planning.

**Clinical relevance statement:**

Ensembles of neural networks (ENN) models could automatically segment renal tumors on routine CTs, enabling and standardizing downstream image analyses and treatment planning. Providing confidence measures and segmentation overlays on images can lower the threshold for clinical ENN implementation.

**Key Points:**

- *Ensembles of neural networks (ENN) segmentation is visualized by color-coded voxelwise tumor probabilities and thresholds*.
- *ENN provided a high segmentation accuracy in internal testing and in an independent external test dataset*.
- *ENN models provide measures of segmentation confidence which can robustly discriminate between successful and inadequate segmentations*.

## Introduction

The annual incidence of renal cell carcinoma in western populations is rising, with a reported increase of kidney cancer incidence from 7 to 11 cases per 100,000 US citizens from 1983 to 2002 [1].

These changes are partially attributable to improved detection of renal tumors due to the widespread use of advanced cross-sectional radiological imaging with increasing incidental detection, in particular of tumors with a diameter of < 4 cm consistent with a T1a stage tumor (small renal masses, SRM) [2]. Smaller-diameter renal tumors are less likely to exhibit hallmark imaging features of malignancy and are more challenging to radiologically characterize regarding their potential malignancy and histological subtype [2]. Due to this diagnostic uncertainty, patients residing in high-scanning regions face higher risks of both partial and total nephrectomy as well as renal ablation, which may reflect overdiagnosis followed by overtreatment [3].

Accurate segmentation of the region of interest (i.e., renal tumors) is an essential preliminary step to conduct quantitative image analyses, such as enhancement analyses or radiomics [4]. Further, renal tumor segmentation may be utilized for volumetry and renal tumor treatment planning [5]. For example, the R.E.N.A.L. nephrectomy score rates the complexity of renal tumors in patients based, among others, on tumor diameter and location, thereby aiding in surgical decision-making and follow-up [6]. Exact renal tumor segmentation could also facilitate the planning of thermal ablation procedures, i.e., to optimize ablation probe placement and ablation margins [7].

Manual tumor segmentation is well established but remains laborious and is prone to high inter-observer variability [8]. This high inter-observer variability requires new methods to learn from an ensemble of trained experts and retain this joined knowledge about coinciding and diverging annotation regions. An automatic renal tumor segmentation algorithm could therefore accelerate and refine the segmentation process, as well as facilitate treatment planning and downstream imaging analyses such as radiomics, ultimately contributing to improved patient care in the setting of renal tumors. In addition, robust renal tumor segmentations could aid in surgical resection as well as treatment planning for renal tumor thermal ablation.

In this study, we thus aim to develop an automatic voxelwise semantic segmentation model for the segmentation of solid renal tumors on contrast-enhanced CT images acquired in clinical routine. Furthermore, we intend to visualize segmentation with different thresholds and associated confidence to promote the transparency and clinical applicability of the proposed algorithm.

## Materials and methods

The local ethics committees at both participating centers gave prior approval to this retrospective study (No 2/4/17 and No 67/19), which is compliant with the Declaration of Helsinki.

### Training dataset

Adult patients with renal tumors referred for surgical resection between 2012 and 2022 at the University Medical Center Goettingen, Department of Urology, and Saarland University, Department of Urology and Pediatric Urology, were included if they received preoperative, contrast-enhanced CT imaging in arterial (corticomedullary) and/or venous (nephrogenic) CM phase. CT scans were performed at the tertiary centers or at outside imaging centers, including hospitals and private practices, without restrictions regarding scanning protocols (i.e., CT scanner type, contrast media amount and administration rates) and image quality, to better reflect clinical heterogeneity and improve the generalizability of the segmentation models.

All CT scans were provided in a 512 × 512 pixel matrix with a median slice thickness of 3 mm (IQR: 1–5 mm). Most patients were examined using Emotion 16 (16.4%) and SOMATOM Definition AS/AS+ scanners (12.5%). Further details regarding specific CT scanners are provided in Supplementary Table 1 (electronic supplementary material).

Analyses were performed irrespective of the diameter and histological subtype of renal tumors. Patients presenting with infiltrative renal tumors (i.e., lymphoma or chronic inflammatory changes), as well as those with cystic renal tumors, were excluded from analyses.

A subset of the training dataset (418 patients) was recently reported in a separate publication on the utilization of radiomic feature analyses for discrimination of renal tumor subtypes (10.1007/s00330-024-10731-6). The study presented here focuses on renal tumor segmentation and adds data on another *n* = 221 patients in the training data, as well as an independent test dataset.

### Test dataset

For independent testing, the publicly available KiTS 2019 challenge dataset was used, including patients with solid renal tumors imaged with CT in the corticomedullary CM phase. All renal tumors in the test dataset were manually segmented by a urological research team at Cornell University, USA [9].

### Renal tumor assessment

Partial or radical nephrectomy specimens from all included renal tumors were histopathologically analyzed at the Department of Pathology at both participating tertiary centers to establish the histological reference standard.

### Radiological renal tumor segmentation

Manual tumor segmentation in the training dataset was performed by an experienced genitourinary radiologist (J.U.; 7 years of experience). All renal tumors were manually segmented on every available axial CT slice with review for consistent segmentations in 3D reconstructions. Manual segmentation was separately performed for arterial and venous CM phases in a blinded fashion. The number of CT slices varied according to the renal tumor size and CT slice thickness: the median number of CT slices containing renal tumors was 13 (IQR: 7–32), as also shown in Supplementary Figs. 1 and 2. The open-source software 3D Slicer was used for renal tumor segmentation.

### Automated renal tumor segmentation

**Fig. 1:**
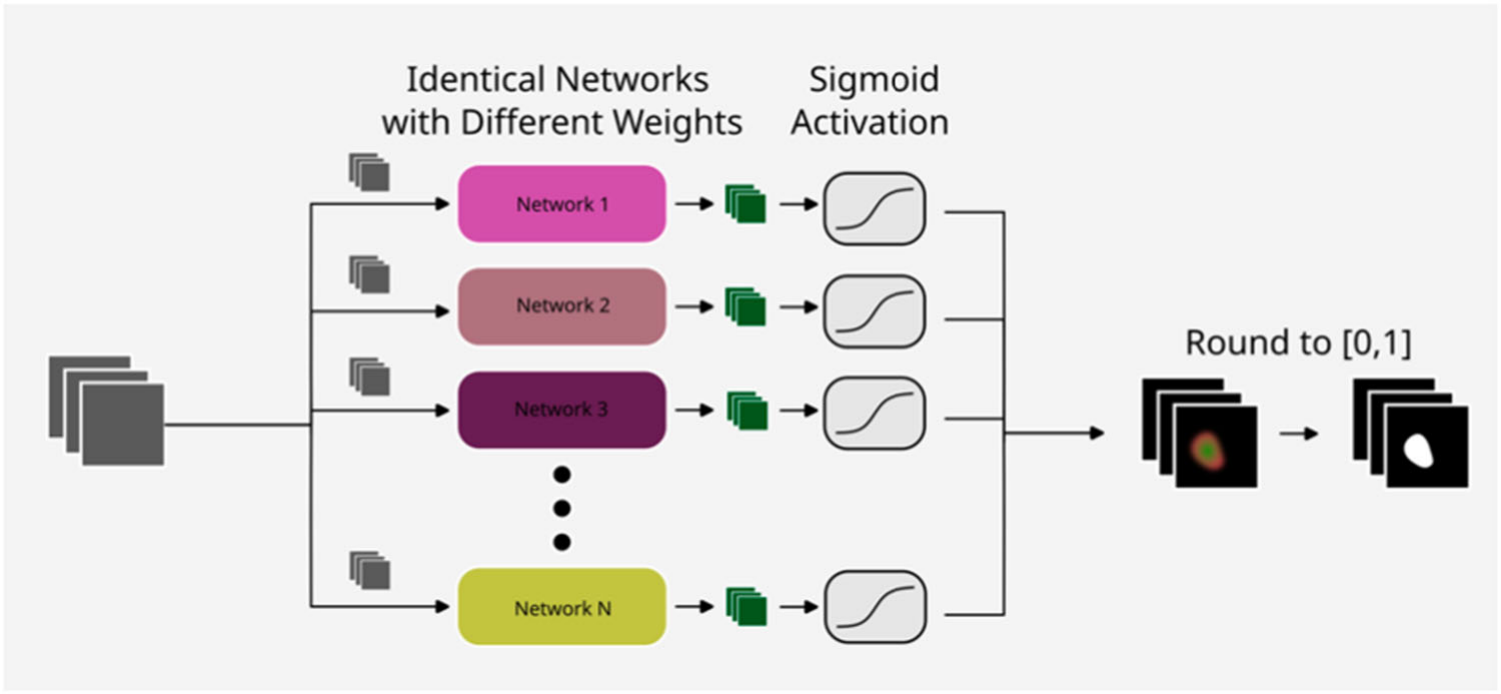
Using ENN, inference is performed multiple times on the same image. To obtain a binary prediction (renal tumor vs. surrounding tissue), the sigmoid outputs are averaged and rounded to either 0 or 1. The combined prediction before rounding is an expression of voxelwise confidence

For the automatic segmentation of renal tumors, ensembles of neural networks (ENN) were trained on all full-body axial CT slices that contained renal tumors, using an existing version of DeepLabV3 based on a ResNet50 backbone available from PyTorch (Model DeepLabV3-ResNet50), where the final classifier was replaced to obtain a binary prediction. Although segmentations are a three-dimensional problem, renal tumor segmentation in this study was treated as independent 2D segmentation tasks on each axial CT slice. Training and inference were performed using the full voxel matrix of 512 by 512 voxels and the full 16-bit color depth. The model was independently trained a total of ten times, resulting in ten different weights (“members” of the ensemble), which were used to predict the same image. After collecting all outputs and applying the sigmoid activation function, the results were averaged and rounded to either 0 or 1 to obtain a binary prediction (renal tumor vs. surrounding tissue) (see Fig. 1).

Each net’s individual set of weights was trained independently, and the distinct variety was introduced by (1) random initialization, (2) randomly shuffling the training images, and (3) random augmentations applied at each iteration. Training was performed for 20 epochs (batches of 64), with a learning rate of 0.1, using Stochastic Gradient Descend (momentum 0.9) and a loss function based on the DICE score adapted from Wolny et al [10].

For the ENN training, the CT imaging data in arterial and venous CM phase from the training dataset (including patients from the University Medical Center Goettingen and Saarland University) was split into three sets for training, validation during training and a hold-out set for internal testing (comprising 80%, 10%, 10% of all CT studies). Care has been taken that datasets coupled to individual patients, i.e., including both CM phases, were in the same dataset to avoid overfitting. The test dataset (independent external KiTS 2019 dataset) was used for testing of the ENN.

### Automated segmentation visualization and confidence

The ENN segmentations were visualized using estimated probabilities of renal tumors ranging from 0 to 100% for each voxel. These probabilities were color-coded with red indicating 0% renal tumor probability, and green indicating 100% renal tumor probability. Color coding was overlaid onto clinical CT scans for each voxel. Additionally, similar tumor probabilities were delineated with contour lines.

To provide clinicians with feedback on the reliability of the ENN segmentation, the ENNs confidence in its own segmentation success was measures. Therefore, the ENNs average foreground entropy was used. In this context, higher entropy measures indicated lower segmentation confidence, i.e., a high probability of a low-quality renal segmentation by the ENN.

Testing of the ENN confidence in its segmentation success was performed on the hold-out sets from the training dataset (10%) and the independent test dataset.

### Statistical analyses

Continuous data were summarized as median and interquartile range (IQR). Categorical data were provided as absolute numbers and percentages. Between the training and test datasets, continuous data were compared using the Wilcoxon rank sum test, and categorical data using the chi-square test.

The DICE score was used to quantify the similarity of the ENN renal tumor segmentation and the reference standard (manual renal tumor segmentation), with a DICE score = 1 indicating a perfect similarity. For the context of this study, a successful segmentation was defined as any segmentation with a DICE score of > 0.8 for the ENN compared to the reference standard and an inadequate segmentation with a DICE score of ≤ 0.8. This dichotomization was based on the expected upper limits of renal tumor DICE score in the literature and potential clinical applicability [11].

The receiver-operating-characteristics curve (ROC-curve) and area-under-the ROC-curve (AUC) were used to evaluate the diagnostic performance of the ENNs confidence in its segmentation success. Sensitivity and specificity of ENN segmentation success were derived from the ROC-curve using the Youden index in the training dataset. All statistical analyses were performed with R version 4.2.1 and Python version 3.10.13. The significance level was set at 0.05. All reported *p*-values are two-sided.

## Results

### Patient cohort

A total of *n* = 639 patients were included in the training dataset, and another *n* = 210 patients in the independent test dataset. Demographic variables, CT imaging technique and tumor parameters are summarized in Table 1.

**Table 1:** Patient demographics, CT technique and renal tumor variables in the training dataset and independent test dataset

	Training dataset	Independent test dataset	*p*-value
Total number of cases	639	210	–
CT contrast media phase*	Corticomedullary *n* = 501 (78%) Nephrogenic *n* = 576 (90%)	Corticomedullary *n* = 210 (100%) Nephrogenic *n* = 0 (0%)	–
Age at diagnosis (years)	Median = 66; IQR = 56–74	Median = 61; IQR = 50–68	0.22
Sex	Female *n* = 233 (36.5%) Male *n* = 406 (63.5%)	Female *n* = 87 (41.4%) Male *n* = 123 (58.6%)	0.44
Histological renal tumor subtype	AML *n* = 31 (4.9%) Chromophobe RCC *n* = 38 (5.9%) Clear-cell RCC *n* = 383 (59.9%) Oncocytoma *n* = 73 (11.4%) Papillary RCC *n* = 86 (13.5%) Other histology *n* = 28 (4.4%)	AML *n* = 5 (2.4%) Chromophobe RCC *n* = 19 (9.0%) Clear-cell RCC *n* = 143 (68.1%) Oncocytoma *n* = 10 (4.8%) Papillary RCC *n* = 21 (10.0%) Other histology *n* = 12 (5.7%)	0.001
Radiological tumor diameter at surgical resection (mm)	Median = 48; IQR = 35–68	Median = 40; IQR = 25–56	< 0.01
Tumor diameter category	0–40 mm *n* = 225 (35.5%) 41–70 mm *n* = 272 (43%) > 70 mm *n* = 136 (21.5%)	0–40 mm *n* = 111 (52.9%) 41–70 mm *n* = 60 (28.6%) > 70 mm *n* = 39 (18.6%)	< 0.01

*AML* angiomyolipoma

* Corticomedullary and nephrogenic contrast media phases not mutually exclusive

### ENN training and internal testing

Training of the ENN was performed on 80% of the training dataset (*n* = 399/*n* = 464 patients in corticomedullary and nephrogenic CM phase with *n* = 11,184/*n* = 13,148 CT slices). Internal testing was performed on 10% of the training dataset (*n* = 46/*n* = 52 patients in corticomedullary and nephrogenic CM phase with *n* = 1176/*n* = 1538 CT slices).

The internal testing yielded a median DICE score of 0.84 (IQR: 0.62–0.97) for the corticomedullary and 0.86 (IQR: 0.77–0.96) for the nephrogenic CM phase, respectively, each compared to the reference standard of manual renal tumor segmentation.

### ENN segmentation: external test on KiTS dataset

For the external independent test of the ENN model, the KiTS dataset with *n* = 210 patients imaged in the corticomedullary contrast media phase was used (*n* = 5712 CT slices). In this dataset with unknown CT images, the ENN achieved a median DICE score of 0.84 (IQR: 0.71–0.97; compared to the reference standard of manual renal tumor segmentation).

### ENN segmentation visualization and confidence

**Fig. 2:**
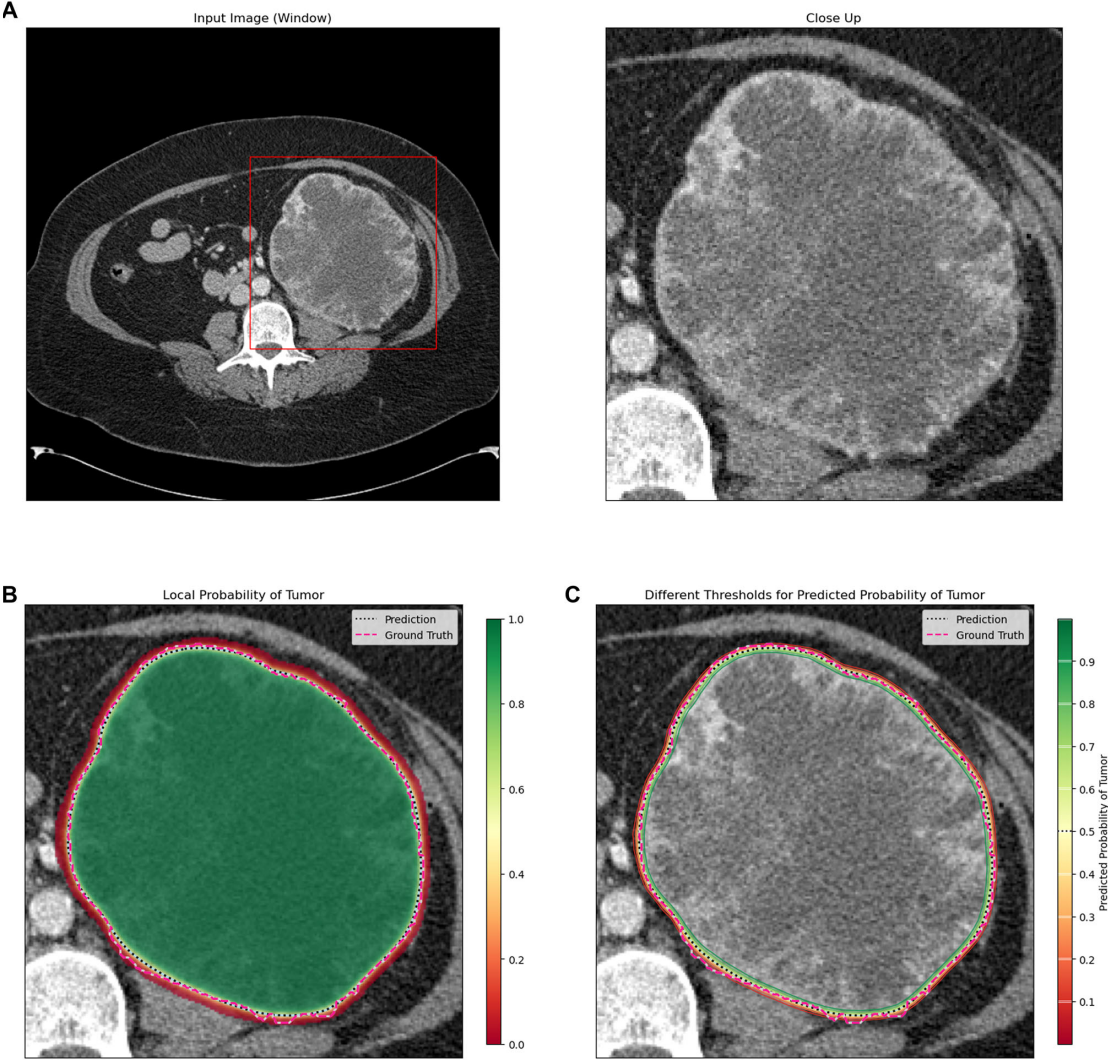
KiTS ID 0067, slice 0202. Case of a 56-year-old female patient with a 13.9 cm diameter left-sided clear-cell RCC (**A**). The ENN segmentation is superimposed as color-coded voxelwise renal tumor probabilities (red: 0% tumor probability; green: 100% tumor probability) and thresholds (contour lines) in **B** and **C**, with a purple line indicating the reference standard (manual segmentation). For this specific CT image, the DICE score = 0.97. The average FG entropy = 0.09 indicated a high confidence of the ENN in its segmentation success

**Fig. 3:**
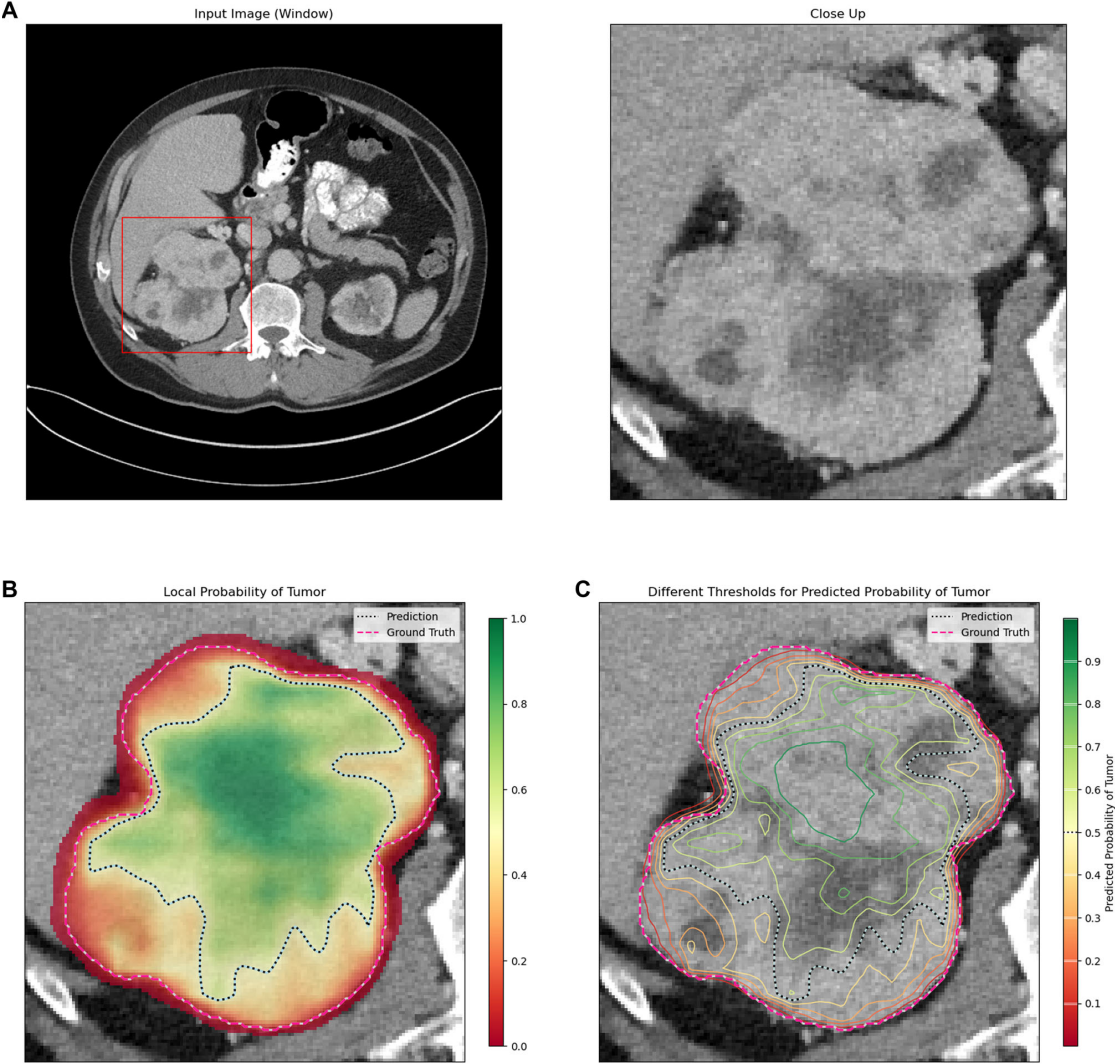
KiTS ID 0008, slice 0125. Case of a 68-year-old male patient with a 10.6 cm diameter right-sided clear-cell RCC (**A**). ENN color-coded voxelwise renal tumor probabilities (red: 0% tumor probability; green: 100% tumor probability) and thresholds (contour lines) in **B** and **C**, with a purple line showing the reference standard, yielding a DICE score = 85. The average FG entropy = 0.42 confirmed a moderate confidence of the ENN in its segmentation success

**Fig. 4:**
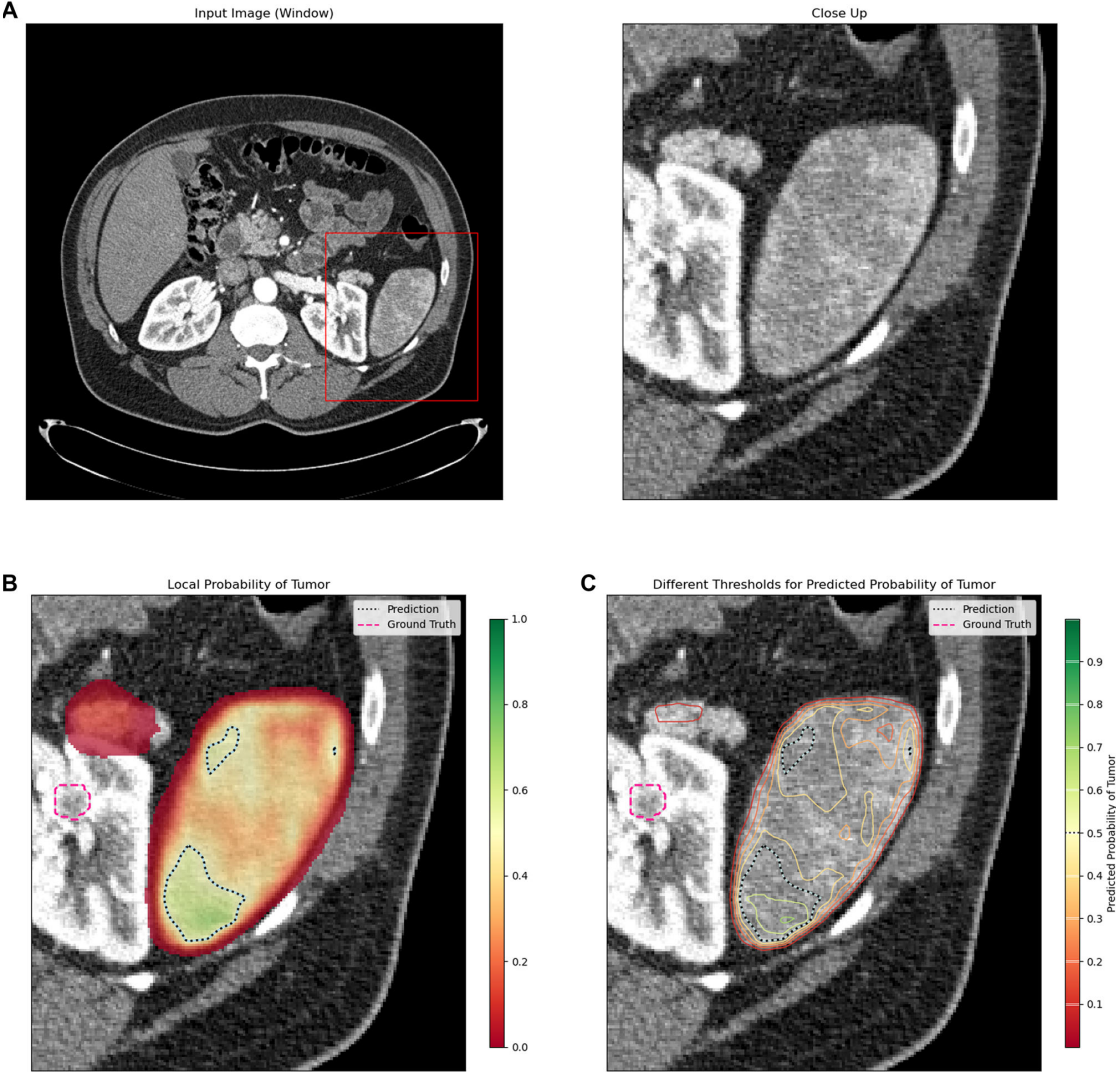
KiTS ID 0140, slice 0150. Case of a 58-year-old male patient with a 2.4 cm diameter left-sided clear-cell RCC (**A**). ENN color-coded voxelwise renal tumor probabilities (red: 0% tumor probability; green: 100% tumor probability) and thresholds (contour lines) in **B** and **C**, with a purple line showing the reference standard, yielding a DICE score = 0 due to erroneous identification of the spleen as renal tumor. An average FG entropy = 0.92 corroborated the low segmentation confidence

ENN segmentations were visualized with color-coding indicating the individual probability of a voxel being renal tumor versus surrounding tissue. Additionally, color-coded probability thresholds were provided. Segmentation probabilities and thresholds (as contour lines) were superimposed on clinical CT images to provide immediate visual feedback, as shown in Figs. 2–4.

**Fig. 5:**
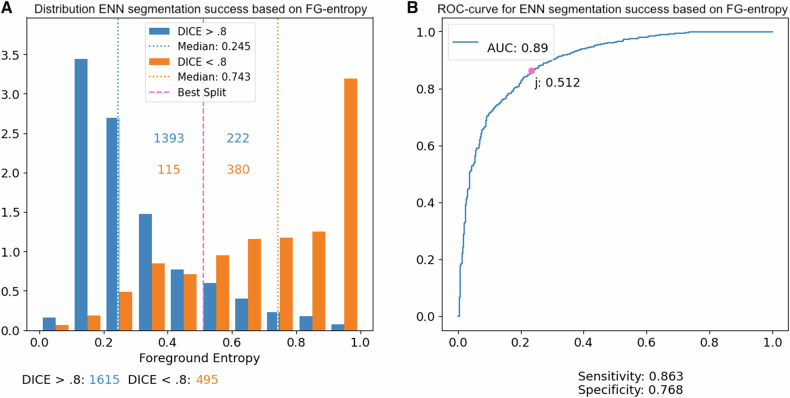
Quantifying the confidence in the ENNs segmentation, a significantly lower median foreground (FG) entropy was evident for CT images with successful segmentation (DICE score > 0.8; median FG entropy = 0.245) versus images with inadequate segmentation (DICE score ≤ 0.8; median FG entropy = 0.743; *p* < 0.001 (**A**); blue indicating successful segmentation and orange inadequate segmentation; absolute numbers of successful and inadequate segmentation CT slices provided below **A**). The corresponding ROC-curve on the hold-out data from the training dataset yielded an AUC = 0.89 (**B**) with an optimal cutoff value at FG entropy = 0.512 for binary discrimination (as determined by the Youden index)

The foreground entropy was used as a measure of the ENNs confidence in its segmentation success, with lower foreground entropy levels indicating a higher confidence. In the training dataset, an AUC = 0.92 was achieved for the discrimination of CT images with successful versus inadequate renal tumor segmentation (cutoff value for foreground entropy = 0.512 based on Youden index; sensitivity = 0.86; specificity = 0.77), as demonstrated in Fig. 5.

**Fig. 6:**
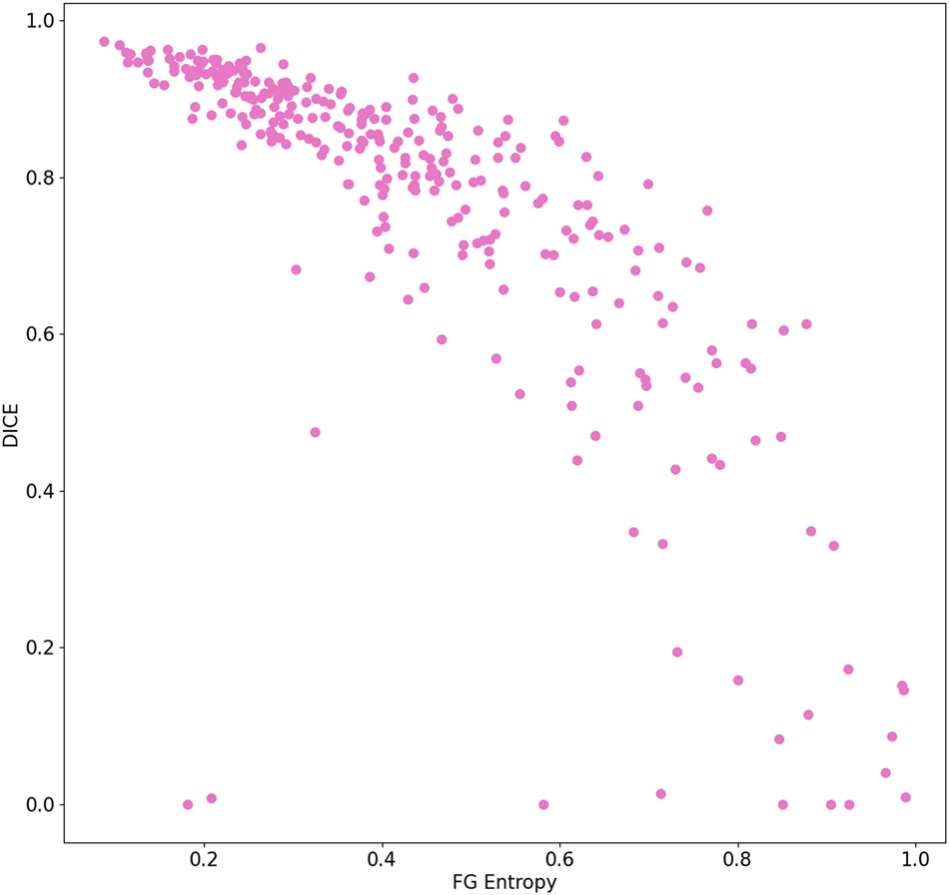
Scatterplot of DICE score per patient and foreground (FG) entropy in the test dataset (*n* = 210). Generally, a higher DICE score is associated with lower FG entropy (indicating higher ENN segmentation confidence). Outliers are observed in the lower right-hand corner where the ENN was confident in its segmentation success (low FG entropy), but the DICE score was low, i.e., due to segmentation of a renal cyst

Applying this foreground entropy threshold on the independent test dataset yielded an accuracy of 0.84, sensitivity = 0.86, and specificity = 0.81. A scatterplot of DICE score and foreground entropy in the test dataset is provided in Fig. 6.

## Discussion

Segmentation of renal tumors is increasingly utilized for volumetry and surgical planning, as well as downstream image analyses, such as radiomic feature analyses [5]. Although manual segmentation of renal tumors on CT images is well established, it remains laborious and is prone to high inter-observer variability between radiologists [8].

In this study, we therefore implemented an ENN to automate and facilitate the segmentation of renal tumors on CT images, visualize their output for radiologists, and provide measures of segmentation confidence.

For the training dataset, we recruited a total of 639 patients with solid renal tumors from two tertiary referral centers in Germany, which is the largest reported renal tumor CT imaging cohort to date [11]. The distribution of age and sex in this training dataset, as well as the external KiTS test dataset, are in line with the literature on renal tumors, demonstrating a male predominance and average age of 60–70 years [12, 13]. Interestingly, differences between the training and test datasets were evident for histological renal tumor subtypes and tumor diameter, with benign tumors (AML, oncocytomas) and larger-diameter tumors being more frequent in the training dataset (both *p* < 0.05). This might be due to differences in center-specific and national guidelines regarding surgical approaches to renal tumors in Europe (training dataset) and the US (test dataset). Also, preoperative biopsies of renal tumors could explain the lower frequency of benign renal tumors in the US-based test dataset. The training dataset in this study was recruited from renal tumor patients undergoing surgical resection, in line with the external test dataset, to obtain a reference standard by histological examination of surgical specimens instead of biopsies alone. This patient selection excluded those with radiologically benign tumors (i.e., fat-rich AMLs) or patients undergoing minimally invasive percutaneous renal tumor treatment, such as microwave ablation or cryoablation. In both tertiary centers, the decision for minimally invasive percutaneous treatment was based on tumor diameter, location, multifocality, comorbidities and the patient’s preference.

Our ENN model achieved good performance for renal tumor segmentation on CT images in the internal validation dataset with a median DICE score = 0.84 in the corticomedullary and 0.86 in the nephrogenic CM phase. This performance was confirmed in the independent external test dataset with a median DICE score = 0.84, although only CTs in the corticomedullary CM phase were available. The reproducible segmentation performance in an independent test dataset acquired in a geographically separate patient cohort with different CT scanners underlines that renal tumor segmentation using ENN is robust and generalizable to external data. Further, our ENN approach demonstrated robust results despite potential systemic differences in the manual renal tumor segmentation on CT images in the training dataset (performed by radiologists) and the test dataset (performed by students under the supervision of urologists). This may be partially attributable to the underlying architecture of ENNs, which are generally known to improve model calibration and performance in deep learning tasks without requiring large changes to the model or training procedures [14, 15]. This facilitates downstream re-training or partial/full switching of the network members. Further, the DICE-based loss ENN training approach applied in our study is known to perform well on imbalanced data commonly encountered in biomedical image segmentations and tends to lead to better performance compared to weighted cross entropy loss [16].

The renal tumor segmentation performance achieved by the ENN in our study is comparable to the literature. For example, using a separate testing dataset of 90 renal tumor patients in the KiTS 2019 segmentation challenge, the highest-ranking teams achieved a DICE score of up to 0.85 using a UNET architecture [11]. Still, the results of this manuscript and the KiTS 2019 challenge are not directly comparable since different test cohorts were used, and the training cohort in this manuscript includes a large-scale external patient group. Further, segmentations in this manuscript were performed by one experienced GU-radiologist as compared to a group of urologists in the KiTS 2019 challenge. Finally, using an ENN approach in this manuscript could have affected the overall results. In summary, the here presented study includes the so far largest patient cohort with utilization of different CM phases and uniquely validated its results in an independent testing dataset.

Our ENN model also provided measures of segmentation confidence, which could accurately discriminate between successful and inadequate segmentations in the training dataset (AUC = 0.89) and the test dataset (accuracy = 0.84, sensitivity = 0.86 and specificity = 0.81). This segmentation confidence could promote and simplify the identification of outlier cases where expert radiologists need to supervise and manually adapt the ENN segmentation. Additionally, it might be used to aid in training resident radiologists, by capturing the knowledge of multiple experienced readers and helping to display predictions of their most likely segmentation decisions for new cases to be discussed with their supervisors.

For ENN segmentation visualization, individual color-coded voxel-prediction values and threshold contour lines were provided and overlaid onto CT images. In contrast to a binary [0/1] output, these linear color-coded segmentation predictions could facilitate the clinical application of ENN models for renal tumor segmentation since radiologists can more easily adjust the ENNs segmentation to individual patients based on their experience. Still, further studies are needed to evaluate the actual usability, acceptance, and impact of ENN segmentations in clinical practice, and how these might be affected by different visualization methods and measures of segmentation confidence.

Our study is not devoid of limitations. First, the test dataset only included patients imaged in the corticomedullary CT CM phase, thus providing no estimate of how well the ENN will perform in the nephrogenic CM phase. Second, there is no true gold standard on the exact delineation of renal tumors at the interface to healthy renal parenchyma, since three-dimensional radiological-pathological mapping of surgical specimens is impossible in a retrospective manner. Thus, while manual renal tumor segmentations can be considered the reference standard, there is inherent associated uncertainty. Third, including only surgically treated patients in this study could potentially limit the generalizability of the ENN segmentation to non-surgical cases, i.e., those patients with fat-rich AMLs or patients referred for thermal ablation. Further, the inclusion criteria of this study might affect the generalizability of findings to the broader population of renal tumor patients encountered in radiological practice. Finally, since only solid renal tumors were included in our study, there is no data on how well our ENN model performs on cystic renal lesions, which needs to be evaluated in separate studies.

## Conclusion

Utilizing a large-scale multicenter CT imaging cohort, we demonstrated that ENN models provide high accuracy for renal tumor segmentation. The ENN model robustly performed on an independent, geographically distinct test dataset with US-based patients, underlining the generalizability of our approach. Providing adaptive, color-coded ENN segmentation predictions as well as measures of ENN segmentation confidence could facilitate the clinical application of the renal tumor segmentation approach. This could aid in renal tumor classification and planning of surgical resection or thermal ablation of renal tumors.

## Supplementary information

ELECTRONIC SUPPLEMENTARY MATERIAL

## Abbreviations

AML: Angiomyolipoma
AUC: Area-under-the receiver-operating-characteristics curve
CM: Contrast media
ENN: Ensembles of neural networks
IQR: Interquartile range
