# Supplementary material for: Renal tumor segmentation, visualization, and segmentation confidence using ensembles of neural networks in patients undergoing surgical resection
Source: Eur Radiol. 2024 Aug 23;35(4):2147–56. doi: 10.1007/s00330-024-11026-6 (PMC11913914; doi:10.1007/s00330-024-11026-6)
Supplement: Supplementary file 1 — ELECTRONIC SUPPLEMENTARY MATERIAL [file 330_2024_11026_MOESM1_ESM.pdf]

Renal Tumor Segmentation, Visualization, and Segmentation Confidence using Ensembles of Neural Networks in Patients Undergoing Surgical Resection

ELECTRONIC SUPPLEMENTARY MATERIAL

Appendix table 1: utilization of CT scanners in the training cohort (n=639)

CT-Scanner	n (%)
Aquilion	54 (8.5%)
Asteion	5 (0.8%)
Astelion	8 (1.3%)
Bright Speed /S	29 (4.5%)
Brilliance 10	1 (0.2%)
Brilliance 16	18 (2.8%)
Brilliance 16P	2 (0.3%)
Brilliance 40	3 (0.5%)
Brilliance 6	50 (7.8%)
Discovery CT750 HD	1 (0.2%)
ECLOS	2 (0.3%)
Emotion 16	105 (16.4%)
Emotion 6	36 (5.6%)
Emotion Duo	2 (0.3%)
GEMINI TF TOF 16	3 (0.5%)
LightSpeed Ultra	3 (0.5%)
LightSpeed VCT	2 (0.3%)
LightSpeed16	2 (0.3%)
MX 16	2 (0.3%)
MX 16-slice	6 (0.9%)
MX 16-Slice	1 (0.2%)
Optima CT520 Series	13 (2.0%)
Optima CT540	8 (1.3%)
Optima CT660	3 (0.5%)
Perspective	10 (1.6%)
Revolution HD	1 (0.2%)
Sensation 10	1 (0.2%)
Sensation 16	6 (0.9%)
Sensation 4	6 (0.9%)
Sensation 40	2 (0.3%)
Sensation 64	1 (0.2%)
Sensation Cardiac 64	5 (0.8%)
SOMATOM Definition	1 (0.2%)
Somatom Definition AS/AS+	80 (12.5%)

SOMATOM Definition Edge	7 (1.1%)
Somatom Definition Flash	64 (10.0%)
Somatom Force	21 (3.3%)
SOMATOM go.Sim	2 (0.3%)
Somatom go.Up	16 (2.5%)
SOMATOM Perspective	7 (1.1%)
Somatom Scope	38 (5.9%)
Spirit	8 (1.3%)
Symphony	1 (0.2%)
Vereos PET/CT	1 (0.2%)
other	2 (0.3%)

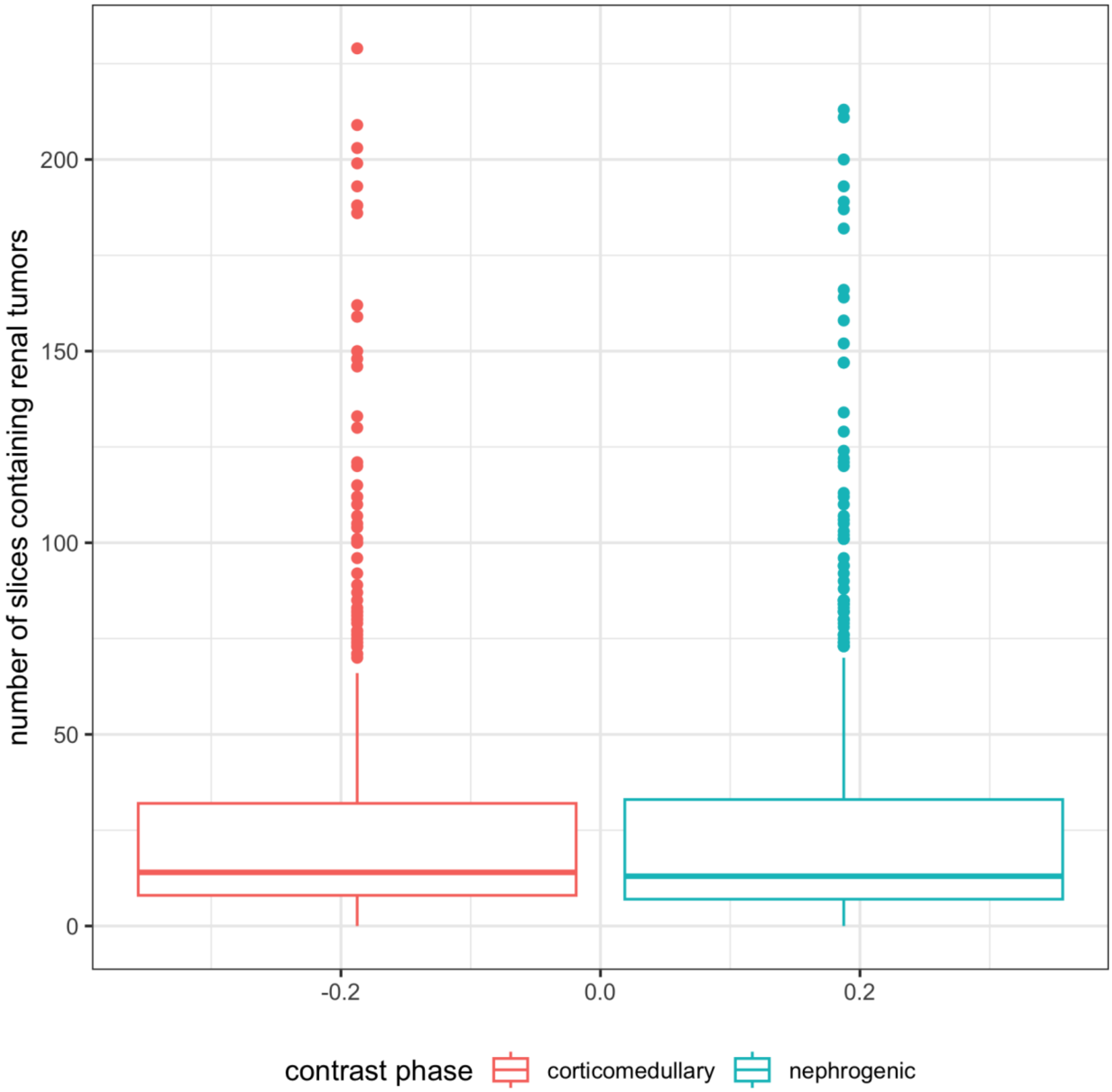

Appendix figure 1: Boxplots of the number of slices per each patient containing renal tumor.

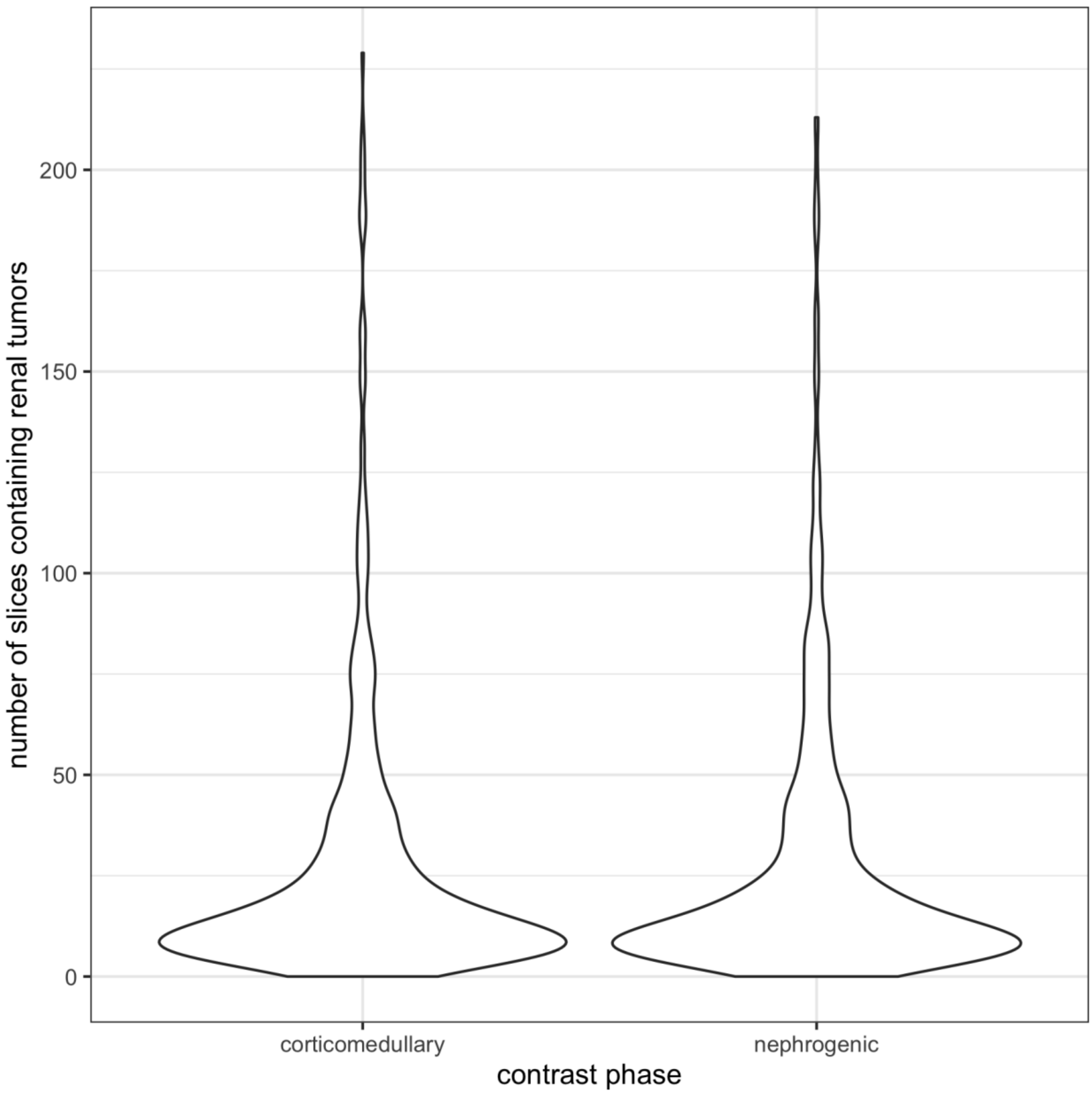

Appendix figure 2: Violin plots of the number of slices per each patient containing renal tumor.
